# A Case Report of a Japanese Boy with Morquio A Syndrome: Effects of Enzyme Replacement Therapy Initiated at the Age of 24 Months

**DOI:** 10.3390/ijms21030989

**Published:** 2020-02-02

**Authors:** Akari Nakamura-Utsunomiya, Toshio Nakamae, Reiko Kagawa, Shuhei Karakawa, Sonoko Sakata, Fumiaki Sakura, Chihiro Tani, Yoshiko Matsubara, Takashi Ishino, Go Tajima, Satoshi Okada

**Affiliations:** 1 Department of Pediatrics, Hiroshima University Graduate School of Biomedical and Health Sciences, Hiroshima 7348511, Japan; 2 Department of Orthopeadic Surgery, Graduate School of Biomedical and Health Sciences, Hiroshima University, Hiroshima 7348551, Japan; 3 Department of Diagnostic Radiology, Hiroshima University Hospital, Hiroshima 7348551, Japan; 4 Department of Diagnostic Radiology, Hiroshima Citizen’s Hospital, Hiroshima 7308518, Japan; 5 Department of Otolaryngology, Hiroshima University Hospital, Hiroshima 7348551, Japan; 6 Division of Neonatal Screening, Research Institute, National Center for Child Health and Development, Tokyo 1578535, Japan

**Keywords:** Morquio A syndrome, mucopolysaccharidosis type IVA, elosulfase alfa, enzyme replacement therapy, growth, spinal decompression surgery

## Abstract

Background: Morquio A syndrome, mucopolysaccharidosis type IVA (MPS IVA), is a lysosomal storage disorder caused by the deficient activity of N-acetylgalactosamine-6-sulfatase (GalNac6S), due to alterations in the *GALNS* gene. This disorder results in marked abnormalities in bones and connective tissues, and affects multiple organs. Here, we describe the clinical course of a Japanese boy with MPS IVA who began enzyme replacement therapy (ERT) at the age of 24 months. Patient: the patient presented for kyphosis treatment at 22 months of age. An X-ray examination revealed dysostosis multiplex. Uronic acids were elevated in the urine and the keratan sulfate (KS) fraction was predominant. The leukocyte GalNac6S enzyme activity was extremely low. The patient exhibited the c.463G > A (p.Gly155Arg) mutation in *GALNS*. Based on these findings, his disease was diagnosed as classical (severe) Morquio A syndrome. An elosulfase alfa infusion was initiated at the age of 24 months. The patient’s body height improved from −2.5 standard deviation (SD) to −2 SD and his physical activity increased during the first 9 months on ERT. However, he gradually developed paralysis in the lower legs with declining growth velocity, which required cervical decompression surgery in the second year of the ERT. The mild mitral regurgitation, serous otitis media, and mild hearing loss did not progress during treatment. Conclusion: early initiation of the elosulfase alfa to our patient showed good effects on the visceral system and muscle strength, while its effect on bones appeared limited. Careful observation is necessary to ensure timely surgical intervention for skeletal disorders associated with neurological symptoms. Centralized and multidisciplinary management is essential to improve the prognosis of pediatric patients with MPS IVA.

## 1. Introduction

Mucopolysaccharidosis type IVA (MPS IVA) (Morquio A syndrome, OMIM #253000) is an autosomal recessive lysosomal storage disease, characterized by the accumulation of keratan sulfate (KS) and chondroitin-6-sulfate, which is caused by homozygous or compound heterozygous mutations in the *GALNS* gene encoding N-acetylgalactosamine-6-sulfatase (GalNac6S) [1]. Because chondroitin-6-sulfate and KS are mainly produced in cartilage [2], the storage of glycosaminoglycan (GAG) within the lysosomes disrupts cell function and metabolism in cartilage [3]; this disruption results in a direct impact on cartilage and bone development, ossification failure, growth imbalance, and unique systemic skeletal dysplasia [4,5,6]. An excessive accumulation of KS in the lysosomes reportedly results in the inhibition of bone formation in osteoblasts [7]. Abnormal chondrogenesis and osteogenesis (dysostosis multiplex) [3] are largely responsible for the typical phenotype of affected patients, including short stature, kyphoscoliosis, prominent bell/barrel-shaped chest, frontal bossing, genu valgum and joint laxity [8,9]. However, intelligence is normal and there is no direct central nervous system involvement, in contrast to the phenotype of other MPS, such as Hurler syndrome and Hunter syndrome. The prevalence of MPS IVA in previous reports (determined using recommended diagnostic methods) ranges from 1 per 71,000 in the United Arab Emirates to 1 per 500,000 in Japan [10].

Elosulfase alfa (Vimizim^®^, BioMarin Pharmaceutical Inc., San Rafael, CA, USA) was approved in Japan in 2015 for use as an ERT for patients with MPS IVA. Previous reports showed that the ERT with elosulfase alfa is well tolerated and produces a reduction in the urine KS [11]. In an initial short-term study, positive effects on growth have been suggested [12]; however, the impact on bone growth has not been proved in the long term (over 2 years) [13,14]. In addition, the clinical information remains limited regarding the clinical course of patients <5 years of age with MPS IVA and their treatment with elosulfase alfa [14]. In this report, we describe the clinical course of a patient with MPS IVA who received the ERT for 25 months, beginning at 2 years of age.

## 2. Patient

The patient was the third boy of non-consanguineous healthy Japanese parents. His two older brothers were both healthy. After an uneventful pregnancy, he was born at a gestational age of 41 weeks by natural delivery; his body weight was 3600g (+1.5 standard deviation [SD]) and his body height was 58cm (+4.3 SD). He exhibited mild hearing loss on the left side during a neonatal screening, and developed serous otitis media in infancy. He demonstrated delayed walking and kyphosis around the age of 12 months. At the age of 16 months, he was examined at the orthopedic department at Hiroshima University Hospital. The examination results were suggestive of a systemic bone disease, therefore, the patient was referred to the pediatrics department at the age of 22 months, with a body height of 78.6 cm (−1.8 SD) and a body weight of 10.86 kg (−0.2 SD). A physical examination revealed no abnormal findings, except lumbar kyphosis, which was most prominent at the level of L1. The patient’s mental development was normal, but his gross motor development was substantially delayed; he could walk alone for approximately 10 m and exhibited a duck-like gait. Routine blood tests did not reveal remarkable findings (Table 1).

## 3. Method

As with the confirmatory tests for MPS IVA, uronic acids in the urine and the enzymatic activity of GalNac6S in the leukocytes were measured by the methods described previously [15]. The results were further confirmed by the direct sequencing of the *GALNS* gene. In brief, all of the exons and flanking intronic regions were amplified using Tks Gflex DNA Polymerase (Takara Bio, Tokyo, Japan). The amplicons were purified using an ExoSAP-IT PCR Product Cleanup kit (Affymetrix Japan, Tokyo, Japan) and sequenced using a BigDye Terminator v3.1 Cycle Sequencing Kit and an Applied Biosystems 3130xl Genetic Analyzer (Thermo Fisher Scientific, Foster City, CA) [15].

## 4. Diagnosis

**Figure 1:**
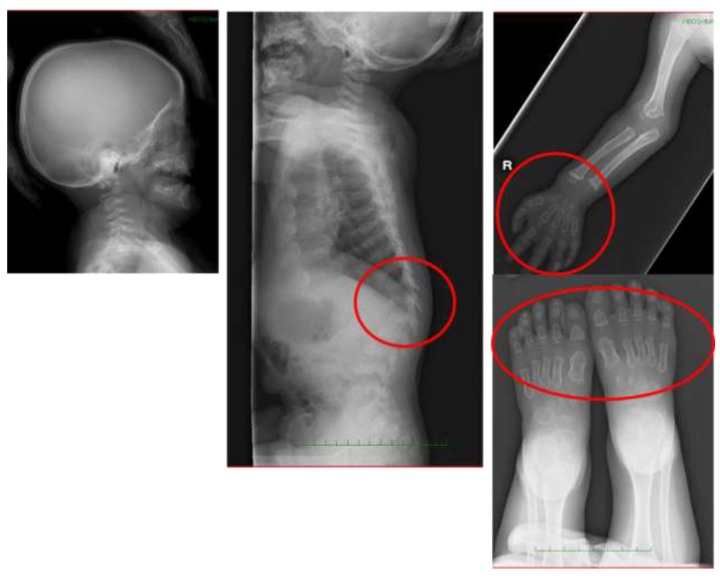
A whole-body X-ray from the first visit of a Japanese boy with MPS IVA. The findings of multiple dysostosis, such as the anterior tongue of the vertebrae and a dumbbell-like deformity in the proximal and middle phalanges of the hands and feet were observed.

**Figure 2:**
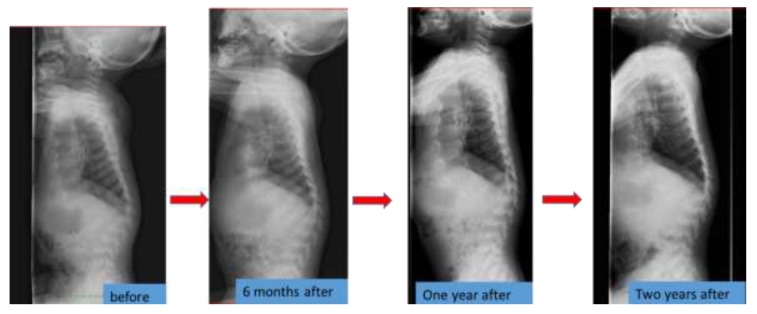
The spine X-ray findings before and after the enzyme replacement therapy in a Japanese boy with MPS IVA.

**Figure 3:**
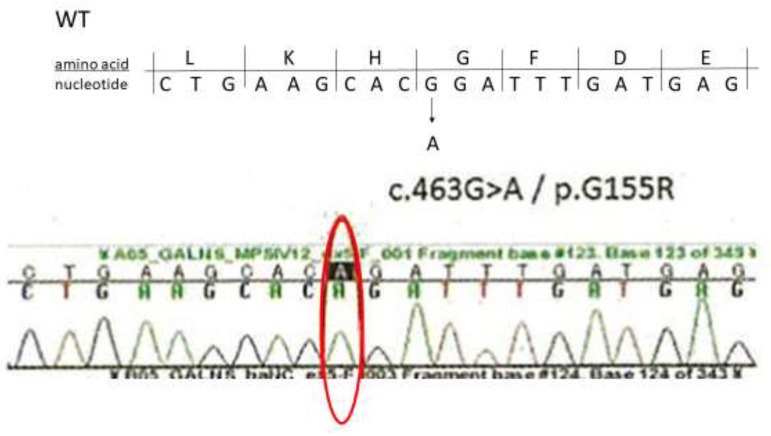
The genetic analysis of the *GALNS* gene in a Japanese boy with MPS IVA. Sanger sequencing of *GALNS* detected a homozygous variant c.463G > A (p.Gly155Arg).

A whole-body X-ray revealed the findings of multiple dysostosis, such as the anterior tongue of the vertebrae and a dumbbell-like deformity in the proximal and middle phalanges of the hands and feet (Figure 1). The spinal magnetic resonance imaging (MRI) showed a moderate compression at C1 level and mild constriction at the thoracolumbar transition (Figure 2). The concentration of uronic acids in the urine was elevated to 166 mg/g·creatinine (reference value: 29.7 ± 13.3 mg/g·creatinine); moreover, a KS fraction as high as 13% was predominant, which indicated MPS IVA. The leukocyte GalNac6S activity was below the determination limit (<1.3 nmol/mg/17 hr) (Figure 3). Sanger sequencing of the *GALNS* gene detected c.463 mcG > A (p.(Gly155Arg)), which was confirmed to be homozygous based on the absence of deletions in microarray analysis (Figure 3). Based on previous reports, this missense mutation is presumed to cause the classical (severe) phenotype of MPS IVA [10].

## 5. Enzyme Replacement Therapy

The enzyme replacement therapy (ERT) with elosulfase alfa (Vimizim^®^, BioMarin Pharmaceutical Inc. (San Rafael, CA, USA); 2 mg/kg weekly) was initiated at the age of 24 months. An oral H1-blocker (Xyzal^®^, GlaxoSmithKline K.K (London, UK); 1.25 mg/dose) was used as premedication to avoid an allergic reaction. A regular administration was performed intravenously for 25 months, with a total of five missed doses; no adverse events were observed. 

## 6. Clinical Findings Before and After Initiation of the ERT

To evaluate the clinical effects of the ERT, the following information was collected: body weight, height and physical activities (monthly), blood tests (bimonthly), a whole body X-ray and spinal MRI (at 6 month intervals); echocardiography and abdominal echography (yearly). The findings are summarized in Table 2.

### 6.1. Urinary GAG Analysis and Other Laboratory Tests

After 12 months of the ERT, the urine KS decreased from 66.24 to 41.71 µg/g·creatinine. The blood KS levels were not evaluated either before or after the ERT. The regular blood tests revealed no abnormal findings, including markers for bone metabolism, such as alkaline phosphatase (ALP) and cross-linked n-telopeptides of type 1 collagen (NTx) in serum. The specific antibodies for the elosulfase alfa (analyzed by BioMarin Japan K.K), after 6 months of administration, showed a positive result of 90.0 dilution factor (normal range, <10), but the neutralizing antibody for the elosulfase alfa remained negative. We also evaluated the patient’s growth hormone (GH) level throughout the follow-up period. The GH level results remained consistently within the normal range, including the arginine-loading dynamic test (peak GH level of 14 ng/mL, normal range ≥ 6 ng/mL).

### 6.2. Body Height and Weight

**Figure 4:**
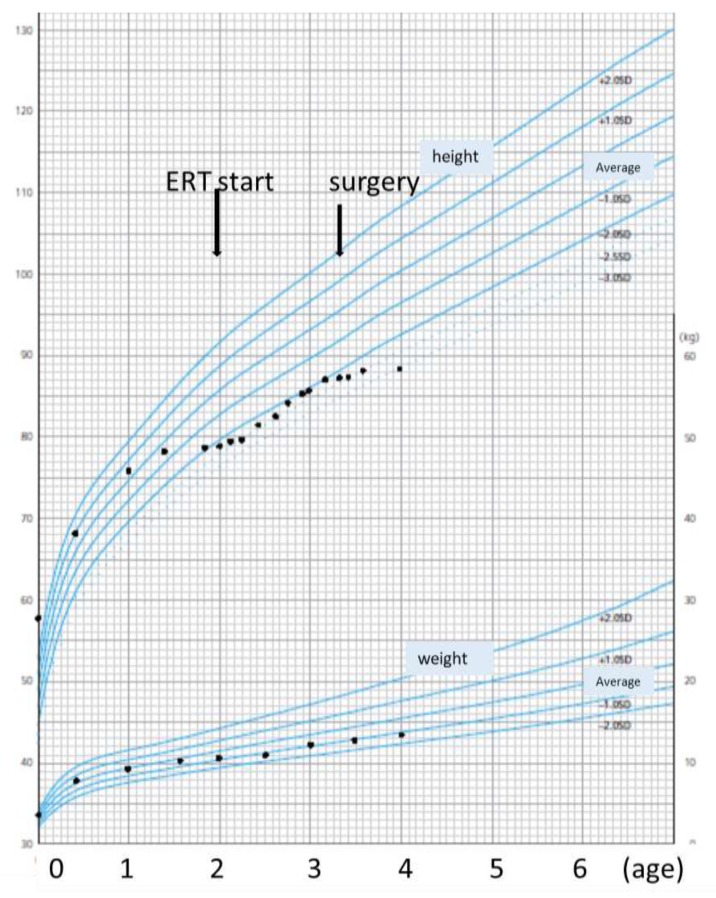
The growth curve of a Japanese boy with MPS IVA. The declining growth velocity of height improved after the initiation of the ERT at the age of 24 months. This chart was referenced from [18].

We measured the patient’s body height in the lying position. He showed a short stature (−2.5 SD on the growth chart for Japanese boys) and a lower velocity of body height (+4cm per year) at the beginning of the ERT. His growth improved to −2 SD on the growth chart after 9 months of treatment—he gained +6 cm during the first year of ERT (Figure 4). Based on a comparison with the growth chart for male patients with MPS IVA [16,17], the patient’s height increased from the 10th percentile to 25th percentile in 1 year. His body weight gained remained at −1 SD. His posture and spinal alignment were both improved in that period. However, the patient’s height gain during the second year on ERT slowed down to 2 cm. 

### 6.3. Physical Activity

At first, the 6 min walk test could not be used to assess our patient. Twelve months after the initiation of the ERT, he could walk 50 m in 6 min, with one hand held by his mother. He was also able to trot, raise his arm higher for ball playing, and carry his backpack by himself. However, in the second year on ERT, he appeared to stumble easily during walking, leading to the use of a walker.

### 6.4. Spinal Lesions

**Figure 5:**
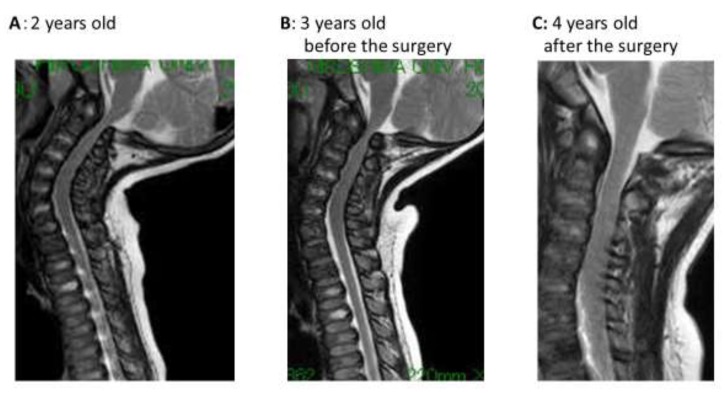
The changes in MRI findings. (**A**) The spinal cord compression at C1 level was already present at the first examination. (**B**) The lesion was slowly progressive after the ERT started, (**C**) which was relieved by resecting the posterior arch of the atlas vertebra.

The spinal cord compression progressed at the level of C1 (Figure 5); the patient gradually developed leg paralysis and ankle clonus during the second year on ERT. At the age of 41 months, a cervical X-ray showed atlantoaxial joint instability, which indicated the need for surgery. We detected the progression of spinal cord compression at the level of C1 in an MRI. The patient gradually exhibited leg paralysis and poor gains in body height and weight in the second year after the ERT. Therefore, we performed the surgery on the cervical spine at 17 months after the ERT. Ordinarily, occipitocervical spine fusion with C1 posterior decompression is performed for patients with MPS IVA. However, as the patient was only 3 years and 5 months of age, the bony structures of the spine were too small to insert the instruments. Accordingly, we decided to first perform the decompression surgery, then perform fusion surgery with instrumentation after the bony structures matured. During general anesthesia, the patient did not exhibit difficulty in securing the airway. Intraoperatively, the posterior arch of C1 was resected, to release the compression of the spinal cord, by using a surgical microscope under the spinal cord for monitoring. The dura mater at the level of compression was covered with fibrous tissue and the resected lesion consisted of cartilage. The decompression findings of the spinal cord were detected by using intraoperative ultrasonography. No complications were detected during the perioperative period. The patient began rehabilitation and continued careful follow-up, as well as the ERT.

### 6.5. Echography and Management in the Other Departments

We performed abdominal and cardiac echography before the ERT and once per year thereafter. Hepatosplenomegaly was not observed. The echocardiography showed trivial mitral valve regurgitation, but no changes before and after the ERT. The serous otitis media and the mild hearing loss that had been present before ERT were improved after the age of 3 years. No complications were present in the ophthalmic and dental evaluations.

## 7. Discussion

Patients with classical MPS IVA exhibit severe skeletal dysplasia and growth disturbance, which affect the respiratory function and cause neurological symptoms, due to the spinal cord compression [19]. The pathophysiology of classical MPS IVA is induced by the accumulation of GAG that is not degraded properly in various organs. The accumulation of GAG reportedly interferes with growth factor production, induces inflammation, and enhances programmed cell death [20]. Systemic skeletal dysplasia and related clinical features are caused by a disruption in the cartilage and its extracellular matrix, leading to a growth imbalance [3,21]. The accumulation of structurally abnormal GAG, such as KS, leads to a direct effect on chondrocyte differentiation [22].

Elosulfase alfa was previously reported to increase the walking distance and muscle strength in patients with MPS IVA. We speculate that the muscle strength improvement by the ERT led to an improved posture and spinal alignment in this patient. These effects were observed in the first year on ERT. However, our patient showed a progression of spine lesions on ERT, which provoked the symptoms of spinal cord compression. It is suggested that the present ERT agent, generally, could reach the bone tissues far less than other visceral organs and tissues. In addition, the worsening of the muscle phenotype may be caused by the low or absent brain tissue targeting. In order to prevent irreversible spinal damage, it is important to evaluate the neurological findings, muscle strength and activities in the patient’s daily life, in addition to a regular spinal MRI. 

Our patient exhibited adequate growth to reach −2 SD during the first year of the ERT. Similar effects of ERT on growth have been reported previously for other patients with MPS of various types. Notably, the growth velocity was poor in the second year, compared with that in the first year. The pathogenesis of short stature in patients with MPS of various types is not completely understood; however, the progressive accumulation of GAG in the cartilage and bones has been identified as the underlying cause of bone abnormalities [23]. Futhermore, GAG storage reportedly induces a complex cascade of secondary and tertiary effects, leading to inflammation and cartilage cell apotosis, thus resulting in a poorly organized and metabolically abnormal connective tissue matrix [24]. In addition, the progression of the severe spinal cord compression in our patient was presumably related to poor growth in the second year because the patient showed a mild loss of appetite with no change in body weight and height, especially in the 3 months before the surgery. We speculate that the progression of the severe spinal compression impaired his appetite, which partially affected his growth in the second year on ERT. Therefore, the combined management with orthopedic surgeons was necessary, despite treatment with the ERT. Moreover, recombinant GH replacement therapy was recently reported to improve body height in a patient with MPS IVA and GH deficiency [25]. We found that GH levels remained within the normal range in our patient. Because it has been reported that patients with MPS IVA exhibit low levels of GH during a long-term clinical follow-up, we will continue to assess the GH level in our patient. A long-term observation is required for the physical growth of the patient.

Our patient tolerated weekly ERT without adverse events, since initiation at as early as 2 years old. A more considerate treatment plan with a shorter infusion time, as well as encouragement of the efforts of the patient and the patient’s family, is important during ERT for younger patients. Monitoring of the elosulfase alfa dose by using the urine KS will facilitate more precise management, although it was performed only once in this patient, where the urine KS decreased by nearly 40% after the ERT. Research is underway regarding new therapies (e.g., pentosane polysulfate) and improvements to existing drugs (e.g., immobilized ERT on nanostructured lipid systems) [26,27]. It is expected that a combination of these drugs can achieve better prognosis for patients with MPS IVA.

## 8. Conclusions

In conclusion, we have described a 2 year old patient with classical MPS IVA. The enzyme replacement therapy was well tolerated and the patient exhibited an improvement in visceral symptoms. However, skeletal disorders and their associated nervous disorders should be carefully evaluated by a multidisciplinary medical team that includes orthopedic surgeons.

## Figures and Tables

**Table 1:** The clinical findings from the first visit of a Japanese boy with mucopolysaccharidosis type IVA (MPS IVA).

	Patients	References
White blood cell count(/µL)	7680	3.3~8.6 × 10^^3^
Red Blood cell count(/µL)	4.58 × 10^^6^	4.35~5.55 × 10^^6^
Hb (g/dL)	11.6	13.7~16.8
PLT(/µL)	327 × 10^^3^	158~348 × 10^^3^
AST(IU/L)	30	13~30
ALT(IU/L)	15	10~42
LDH(IU/L)	239	124~222
BUN(mg/dL)	17.3	8~20
Cr,(mg/dL)	0.20	0.65~1.07
Na(mEq/L)	139	138~145
K(mEq/L)	4.4	3.6~4.8
Cl(mEq/L)	107	101~108
Ca(mg/dL)	5.0	4.3~5.2
P(mg/dL)	4.9	2.5~4.7
CRP(mg/dL)	0.02	<0.3
ph (vein)	7.484	7.35~7.45
pCO2	27.4	35~45
HCO3-	20.3	22~24
BE	−0.7	−3.0~3.0
		
Leukocyte GalNac6S Activity(nmol/mg protein/17 h)	<1.3	104.6
Urine uronic acid (mg/g·creatinine)	166	29.7 ± 13.3
Fraction of KS (%)	13	Not detected

**Table 2:** The clinical course of a Japanese boy with MPS IVA.

	Before	6 Months after ERT	12 Months after ERT	25 Months after ERT
Body height	79 cm (−2.3 SD)	81 cm (−2.5 SD)	85.5 cm (−2 SD)	88 cm (−2.9 SD)
Urine KS(µg/g·creatinine)	66.24	-	41.71	-
Walk	4~5 steps	Walk longer and Trot	50 m for 6 min	By walker
Posture at sitting	A forward-bend posture	Up and Better posture	Keep better posture	Slight forward tilting posture
Kyphosis angle (degree)	126	131	141	138
Shoulder joint mobility	Disability of elevation	Improve of elevation (ball playing)	Keep elevation (shoulder backpack)	Keep elevation
Spinal compression at foramen magnum	Compression by magnetic resonance imaging (MRI) No symptom	-	Compression by MRI No symptom	Improvement after decompression surgery
Echocardiography	MR trivial	MR trivial	MR trivial	MR trivial
Abdominal echography	Hepatosplenomegaly-	-	-	-
Otolaryngeography	Otitis media + Mild deafness(40Db)	Otitis media+	Otitis media- Improved	No medication Hearing test: normal(25Db)
Opthalmology	Not particular	-	Not particular	Not particular
Communication level	Several words	Short talk	Talk well	Communicate well

Abbreviations: mitral valve regurgitation (MR).

## Abbreviations

N-acetylgalactosamine-6-sulfatase	GalNac6S
MPS	mucopolysaccharidosis
GAG	glycosaminoglycan
KS	keratan sulfate
ERT	enzyme replacement therapy
SD	standard deviation
MRI	magnetic resonance imaging
GH	growth hormone
